# Supplementary material for: Potential global impacts of alternative dosing regimen and rollout options for the ChAdOx1 nCoV-19 vaccine
Source: Nat Commun. 2021 Nov 4;12:6370. doi: 10.1038/s41467-021-26449-8 (PMC8569205; doi:10.1038/s41467-021-26449-8)
Supplement: Supplementary file 1 — Supplementary Information [file 41467_2021_26449_MOESM1_ESM.pdf]

Supplementary Fig. 1: Impact of vaccine delivery speed, dose split, and dose availability on vaccine effectiveness as a measure of reduction in clinical cases.

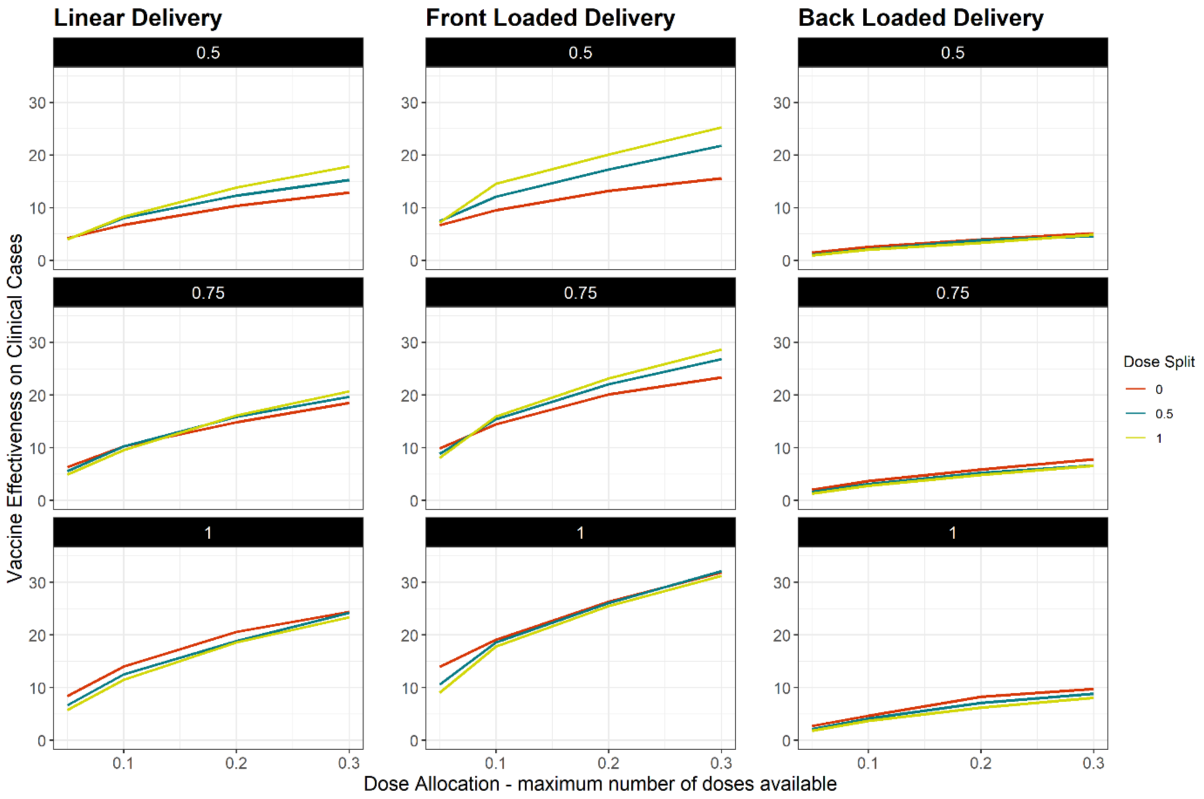

The white number on the black background in each panel defines the vaccine efficacy of the first dose relative to the second dose. Lines represent the mean effectiveness calculated using all runs, where the parameters are those defined by each figure, irrespective of all remaining parameters. Lines are coloured according to the dose split, i.e. the proportion of individuals receiving two vaccine doses. These results are based on the UK population structure.

Supplementary Fig. 2: Impact of vaccine delivery speed, dose split, and dose availability on vaccine effectiveness as a measure of reduction in deaths.

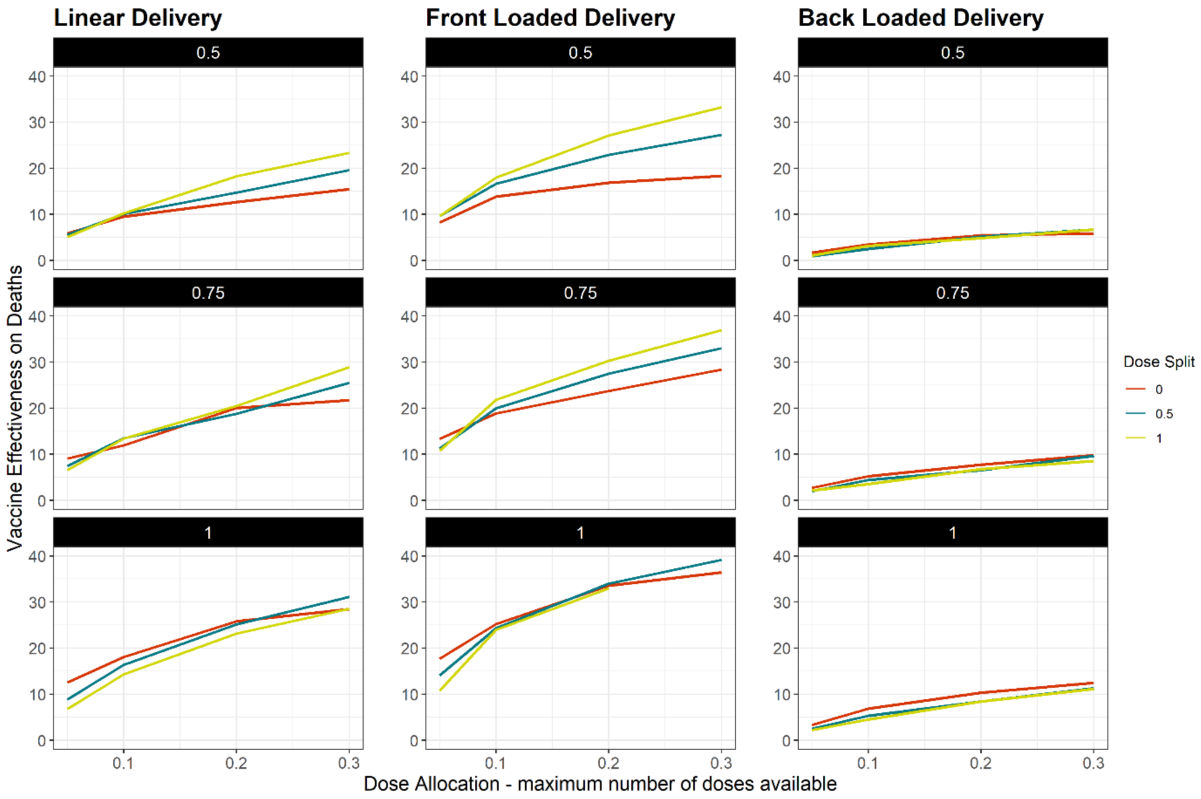

The white number on the black background in each panel defines the vaccine efficacy of the first dose relative to the second dose. Lines represent the mean effectiveness calculated using all runs, where the parameters are those defined by each figure, irrespective of all remaining parameters. Lines are coloured according to the dose split, i.e. the proportion of individuals receiving two vaccine doses. These results are based on the UK population structure.

Supplementary Fig. 3: Detailed sensitivity analysis of vaccine effectiveness for the most sensitive parameters, based on UK data.

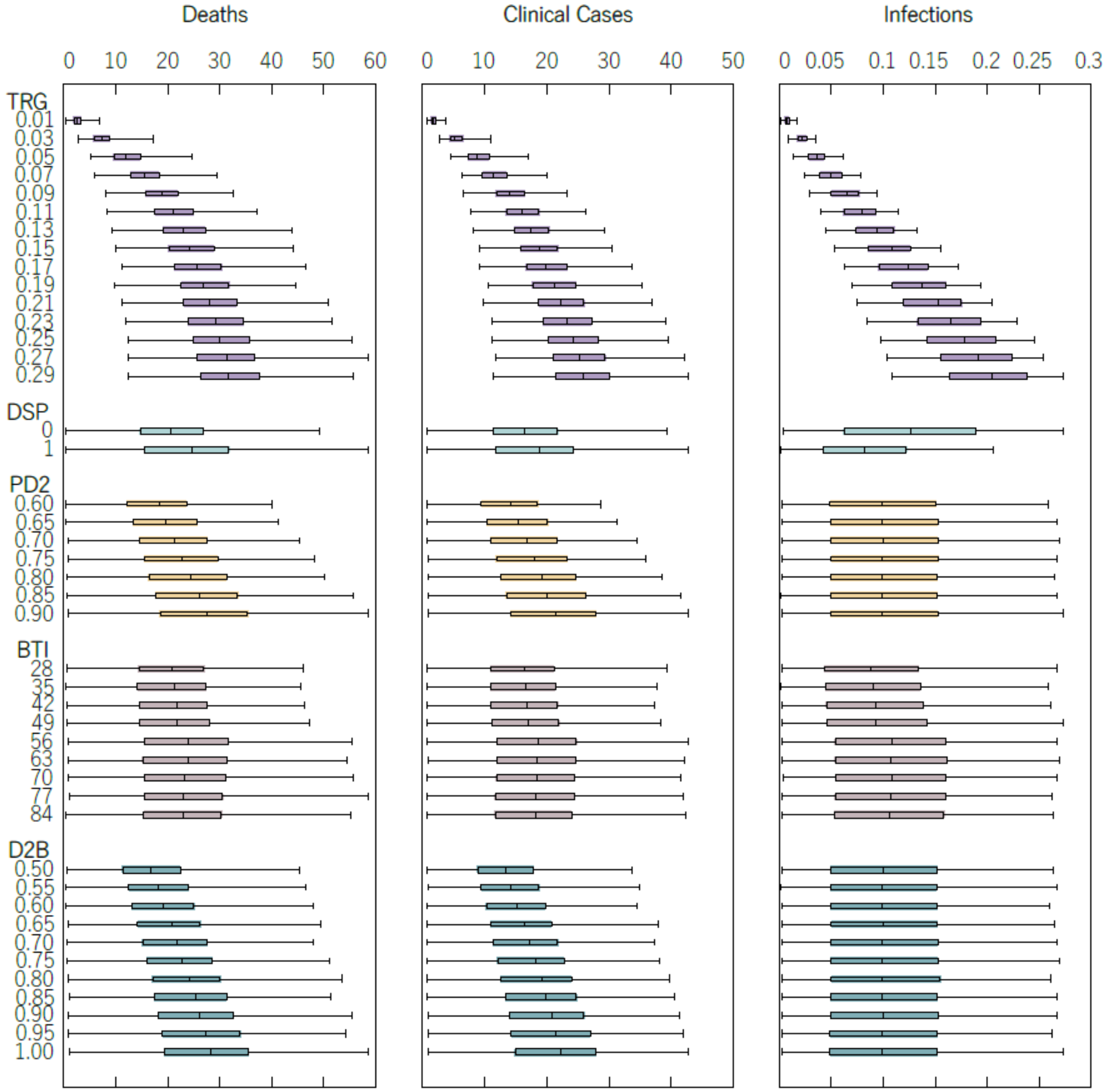

The boxplots show the median and interquartile ranges of the predicted vaccine effectiveness on each of the outcomes for specific parameters. They were generated by aggregating all model simulations for each of the parameters, with each boxplot summarizing the variance in predicted vaccine efficacy for all possible combinations of the other parameters. The middle line shows the median, the lower and upper hinges correspond to the first and third quartiles, and the whiskers extend to the 5th and 95th percentiles. TRG - Vaccine allocation (% of the population during study period); DSP - Second dose administered (% of the vaccinated population administered a second dose); PD2 - Vaccine efficacy after the second dose; BTI - Interval between first dose and booster dose; D2B - Vaccine efficacy of the first dose compared with the second dose (%).

Supplementary Fig. 4: Impact of vaccine booster dose interval on vaccine effectiveness as a measure of reduction in clinical cases.

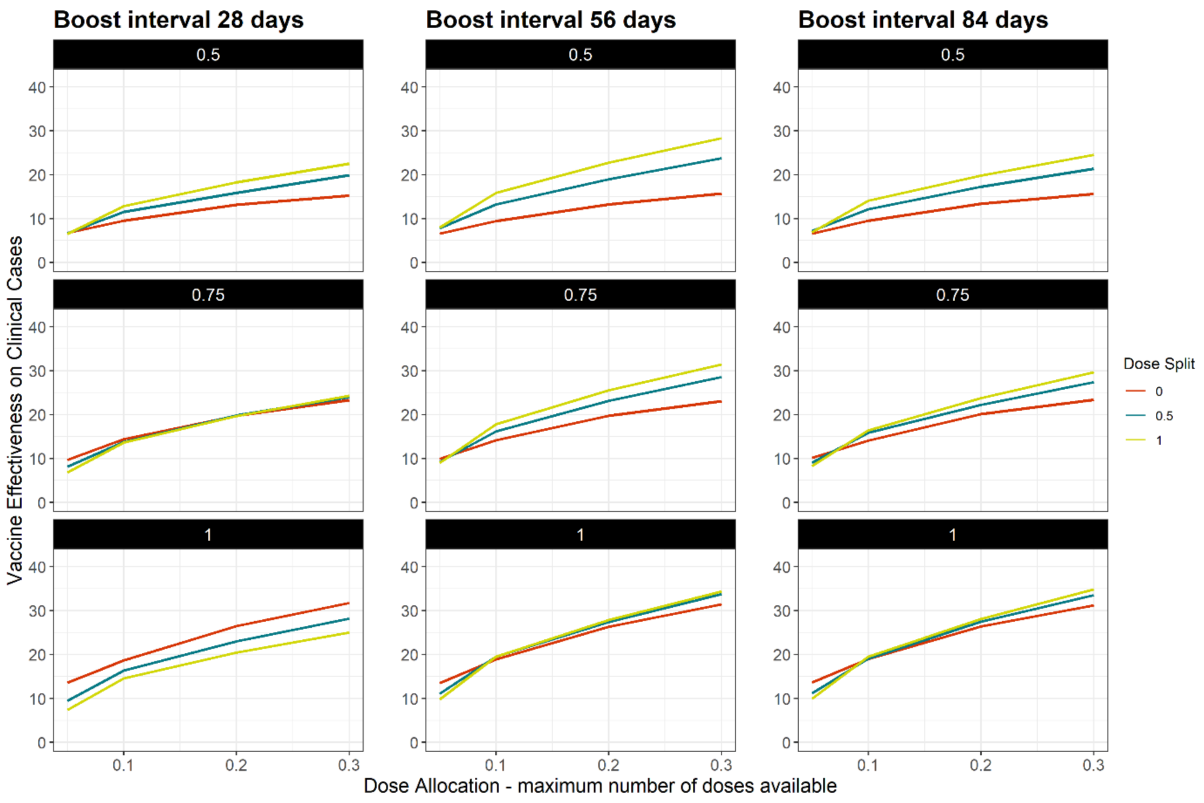

The white number on the black background in each panel defines the vaccine efficacy of the first dose relative to the second dose. Lines represent the mean effectiveness calculated using all runs, where the parameters are those defined by each figure, irrespective of all remaining parameters. Lines are coloured according to the dose split, i.e., the proportion of individuals receiving two vaccine doses. These results are based on the UK population structure.

Supplementary Fig. 5: Impact of vaccine booster dose interval on vaccine effectiveness as a measure of reduction in deaths.

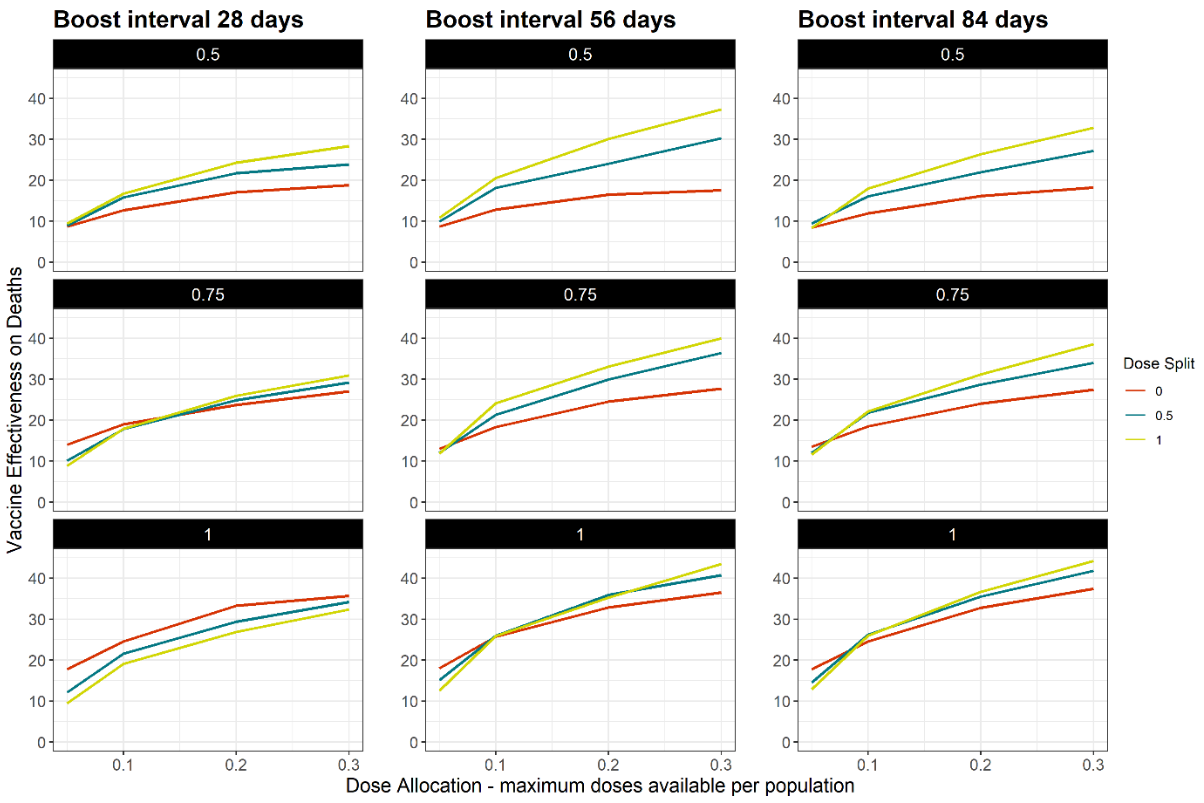

The white number on the black background in each panel defines the vaccine efficacy of the first dose relative to the second dose. Lines represent the mean effectiveness calculated using all runs, where the parameters are those defined by each figure, irrespective of all remaining parameters. Lines are coloured according to the dose split, i.e., the proportion of individuals receiving two vaccine doses. These results are based on the UK population structure.

Supplementary Fig. 6: Vaccine delivery speed.

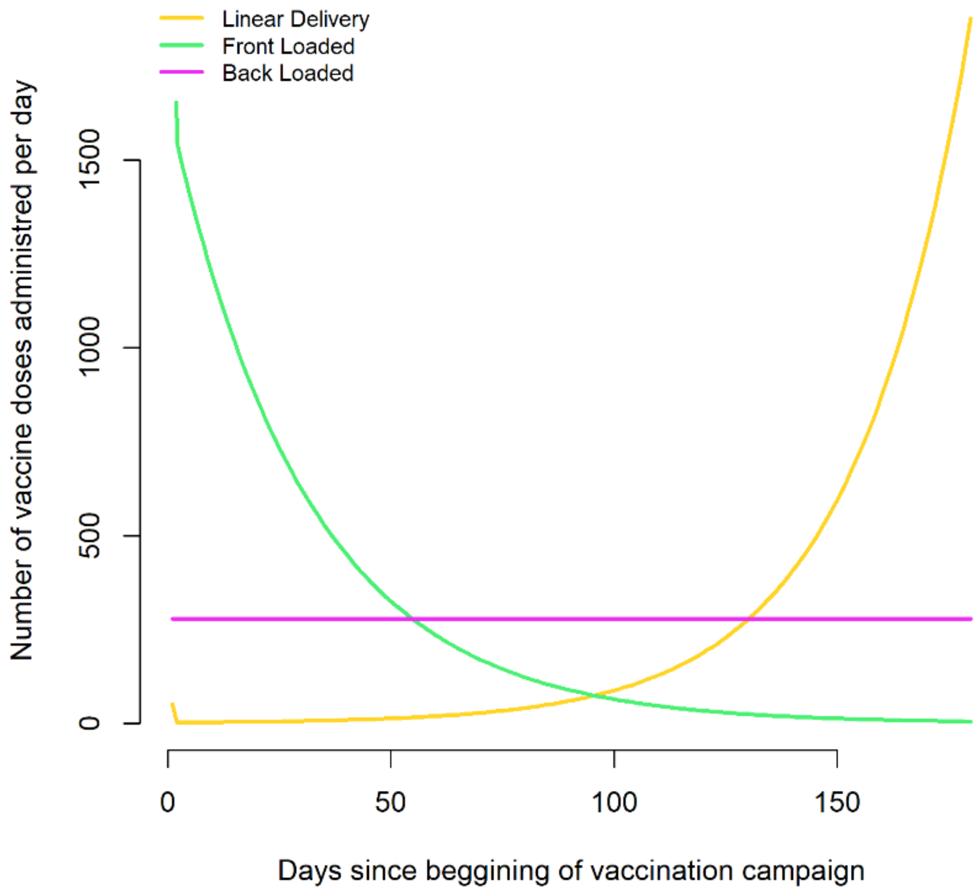

The figure shows the number of vaccine doses administered over the course of the vaccination campaign (6 months) for each of the explored delivery strategies.

Supplementary Fig. 7: Expected vaccine induced decrease in deaths.

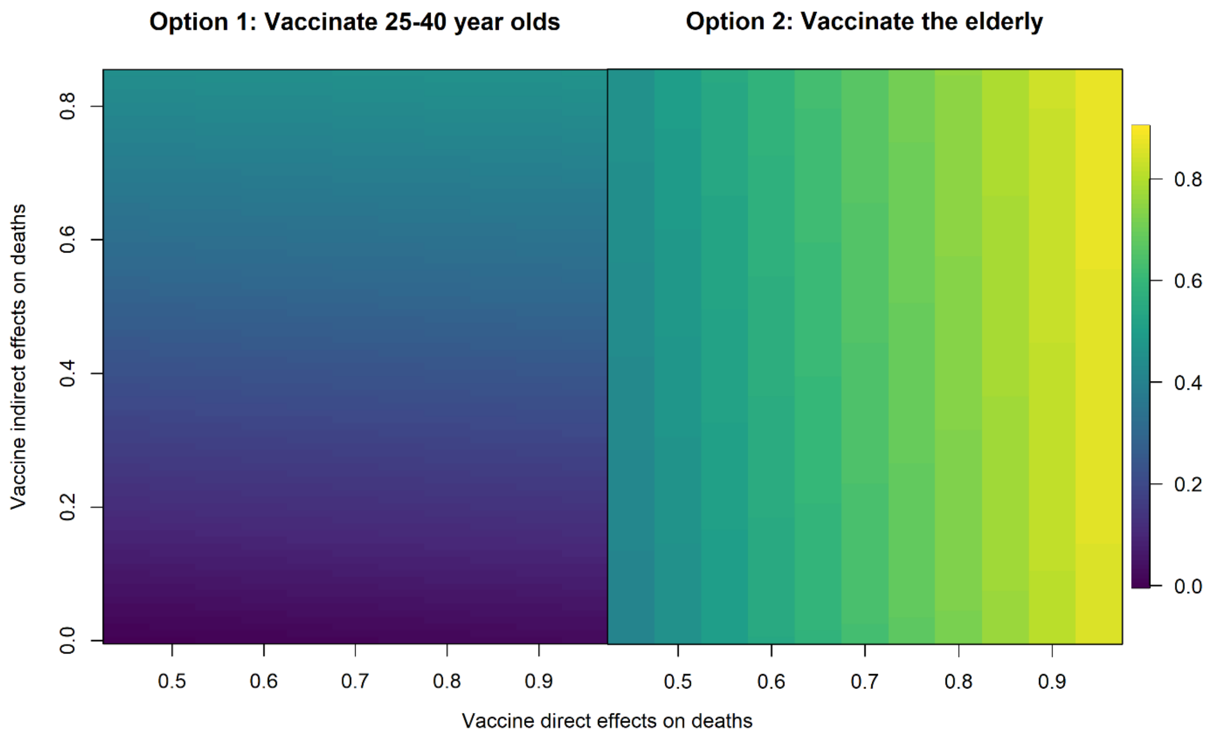

The vaccine induced decrease in the expected number of deaths in a theoretical population where 100,000 infections would occur over 6 months, if no vaccines where given, is presented as a proportion indicated in the colour bar.

Supplementary Fig. 8: Expected vaccine induced decrease in infections.

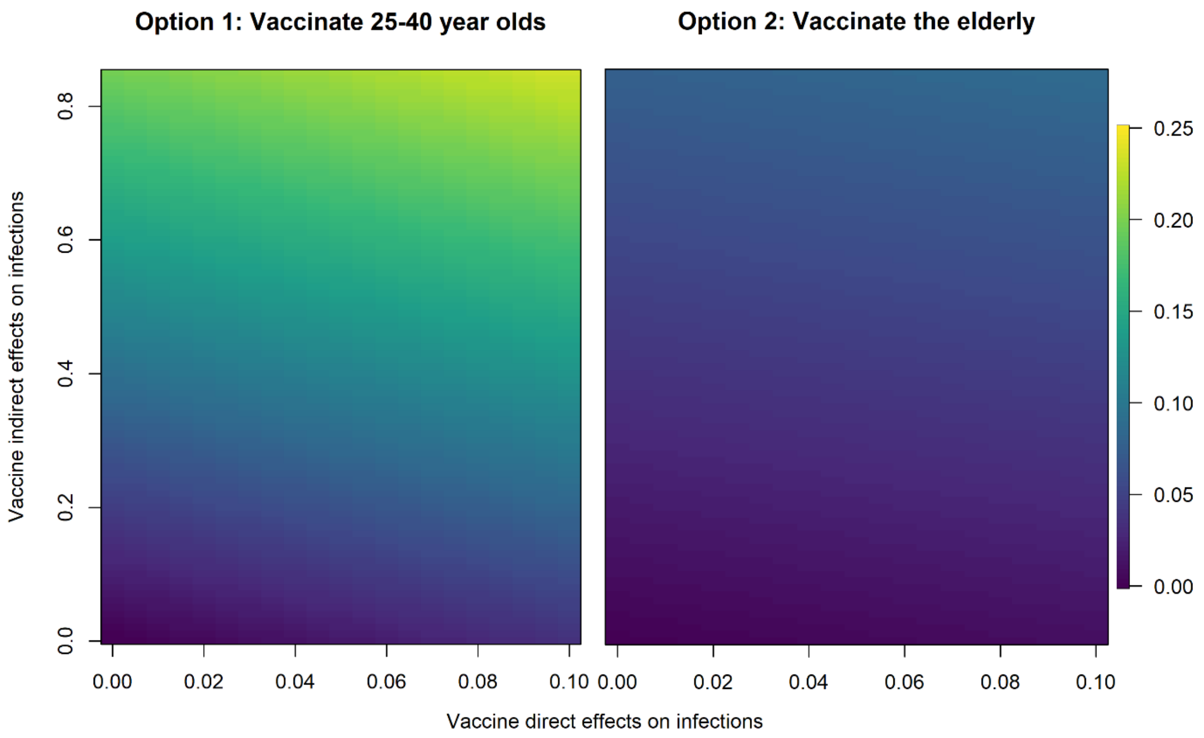

The vaccine induced decrease in the expected number of infections in a theoretical population where 100,000 infections would occur over 6 months, if no vaccines where given, is presented as a proportion indicated in the colour bar.

Supplementary Fig. 9: Simulated delay between first and second dose in our synthetic population.

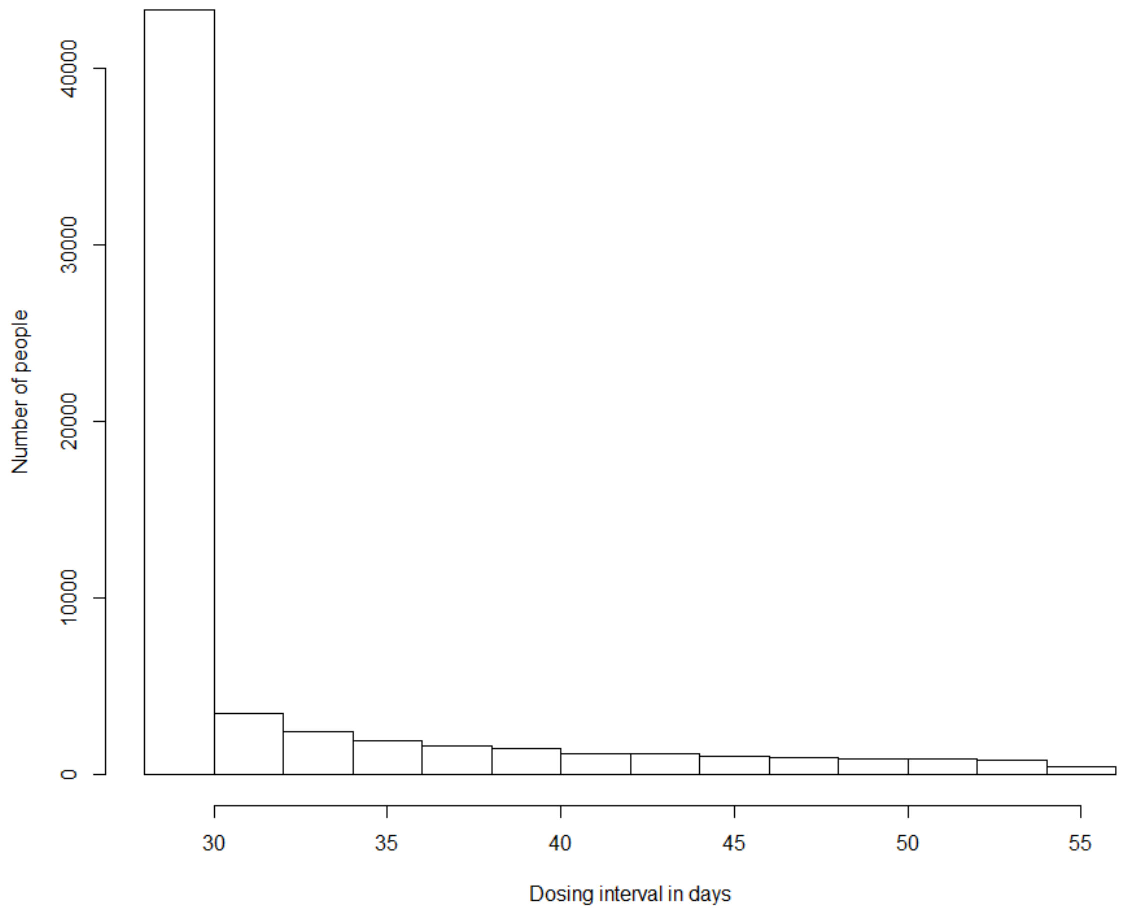

The histogram illustrates the time delay beta function for the 4-week dose interval simulations.

Supplementary Fig. 10: Detailed sensitivity analysis of vaccine effectiveness for the most sensitive parameters, based on UK data, assuming that vaccines have a 25% to 50% impact on onwards transmission (RT).

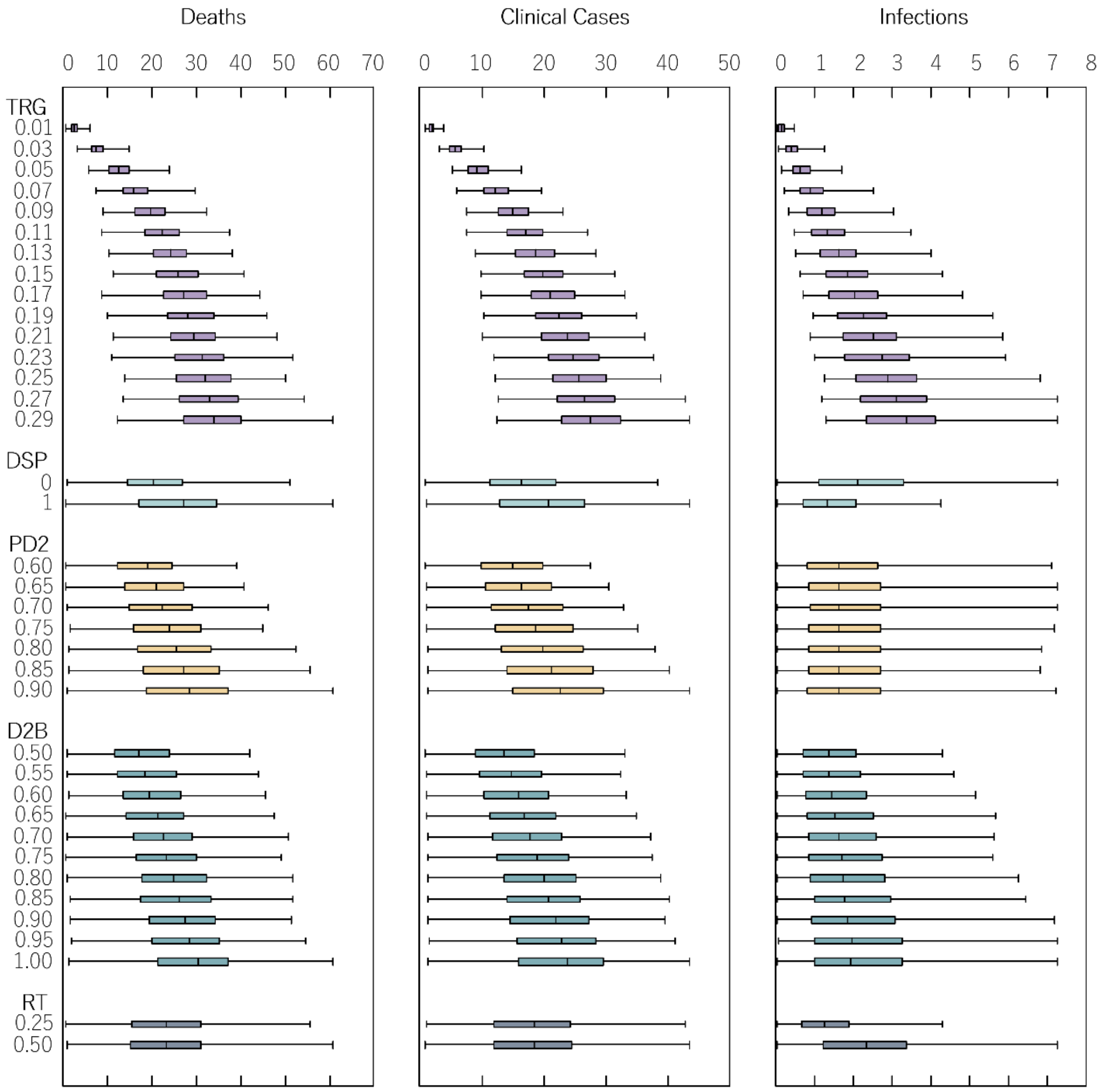

The boxplots show the median and interquartile ranges of the predicted vaccine effectiveness on each of the outcomes. They were generated by aggregating all model simulations for each of the parameters, with each boxplot summarizing the variance in predicted vaccine efficacy for all possible combinations of the other parameters. The middle line shows the median, the lower and upper hinges correspond to the first and third quartiles, and the whiskers extend to the 5th and 95th percentiles. TRG - Vaccine allocation (% of the population during study period); DSP - Second dose administered (% of the vaccinated population administered a second dose); PD2 - Vaccine efficacy after the second dose; D2B - Vaccine efficacy of the first dose compared with the second dose (%); RT - Vaccination impact on the effective reproduction number.

Supplementary Fig. 11: Dose allocation thresholds for the UK assuming different vaccine impacts on transmission.

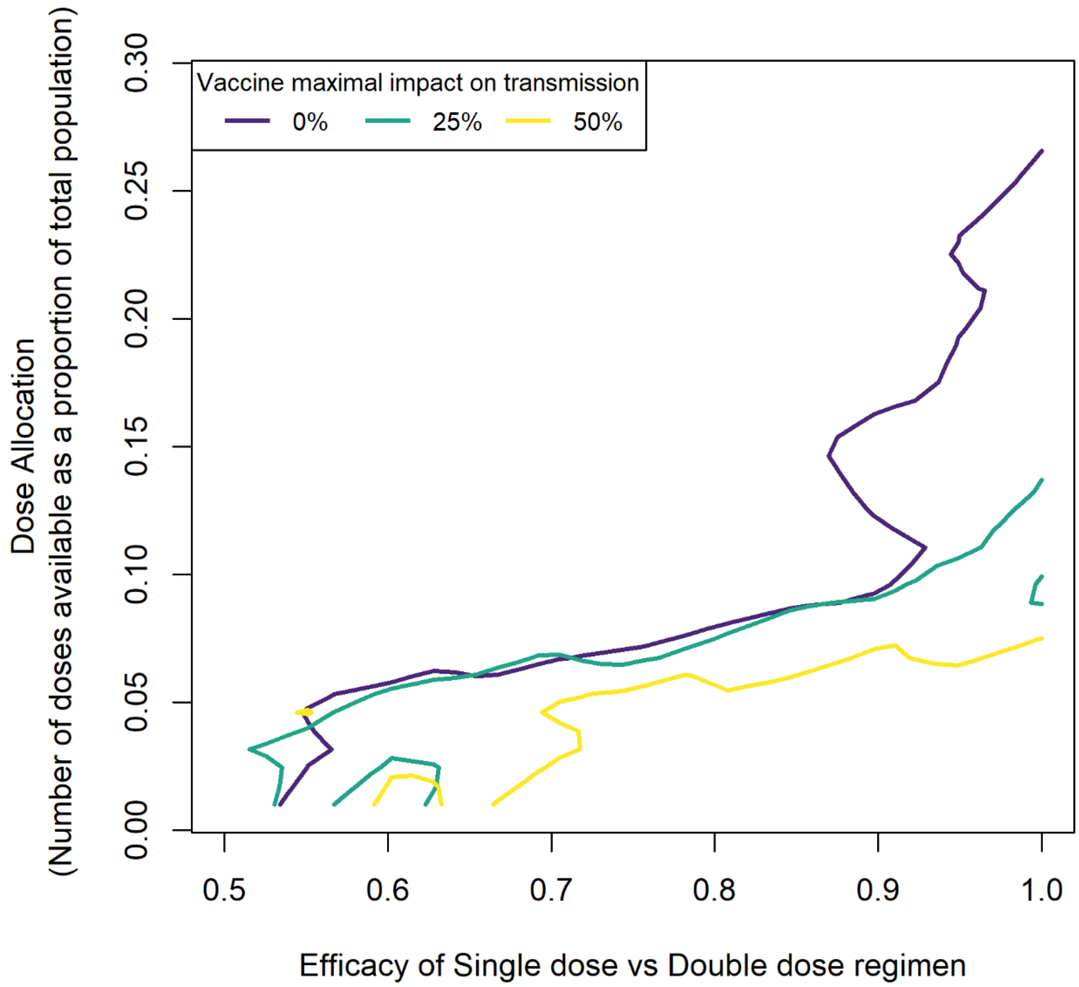

The figure illustrates the parameter combinations that define the allocation threshold above which a two-dose regimen would be preferred over a single-dose regimen.
